# Italian natural history museums on the verge of collapse?

**DOI:** 10.3897/zookeys.456.8862

**Published:** 2014-11-24

**Authors:** Franco Andreone, Luca Bartolozzi, Giovanni Boano, Ferdinando Boero, Marco A. Bologna, Mauro Bon, Nicola Bressi, Massimo Capula, Achille Casale, Maurizio Casiraghi, Giorgio Chiozzi, Massimo Delfino, Giuliano Doria, Antonio Durante, Marco Ferrari, Spartaco Gippoliti, Michele Lanzinger, Leonardo Latella, Nicola Maio, Carla Marangoni, Stefano Mazzotti, Alessandro Minelli, Giuseppe Muscio, Paola Nicolosi, Telmo Pievani, Edoardo Razzetti, Giorgio Sabella, Marco Valle, Vincenzo Vomero, Alberto Zilli

**Affiliations:** 1 Museo Regionale di Scienze Naturali, Via G. Giolitti, 36, I-1023 Torino, Italy; 2 Museo di Storia Naturale, Sezione Zoologica “La Specola”, Via Romana, 17, I-50125, Firenze, Italy; 3 Museo Civico di Storia Naturale, Via San Francesco di Sales, 188, I-10022 Carmagnola, Italy; 4 DiSTeBa - Museo di Biologia Marina “Pietro Parenzan” - Università del Salento, I-73100 Lecce, Italy; 5 Università Roma Tre, Dipartimento di Scienze, Viale G. Marconi, 446, I-00146 Roma, Italy; 6 Museo di Storia Naturale, Santa Croce, 1730, I-30135 Venezia, Italy; 7 Museo Civico di Storia Naturale, Via Dei Tominz, 4, I-34139 Trieste, Italy; 8 Museo Civico di Zoologia, Via U. Aldrovandi, 18, I-00197 Roma, Italy; 9 Università di Sassari, Dipartimento di Scienze della Natura e del Territorio, Zoologia, Via F. Muroni, 25, I-07100 Sassari, Italy; 10 Università degli Studi di Milano-Bicocca, ZooPlantLab, Dipartimento di Biotecnologie e Bioscienze, Piazza della Scienza, 2, I-20126 Milano, Italy; 11 Museo Civico di Storia Naturale, Corso Venezia, 55, I-20121 Milano, Italy; 12 Museo di geologia e paleontologia, Dipartimento di Scienze della Terra, Via Valperga Caluso, 35, 10125 Torino, Italy; 13 Museo Civico di Storia Naturale “G. Doria”, Via Brigata Liguria, 9, I-16121 Genova, Italy; 14 Museo di Storia Naturale del Salento, S.P. Calimera-Borgagne, km 1, I-73021 Calimera, Italy; 15 Science journalist – Swim (Science writer in Italy), Via Jannozzi, 8, I-20097 San Donato Milanese, Italy; 16 Viale Liegi, 48, I-00198 Roma, Italy; 17 MUSE Museo delle Scienze, Corso del lavoro e della scienza, 3, I-80123 Trento, Italy; 18 Museo Civico di Storia Naturale, Lungadige Porta Vittoria, 9, I-37129 Verona, Italy; 19 Dipartimento di Biologia, Università degli Studi di Napoli Federico II, Via Cinthia, 21, I-80126 Napoli, Italy; 20 Museo di Storia Naturale, Via F. de Pisis, 24, I-44100 Ferrara, Italy; 21 Università di Padova, Dipartimento di Biologia, Via U. Bassi, 58/B, I-35131 Padova, Italy; 22 Museo Friulano di Storia Naturale, Via Marangoni, 39 – 41, I-33100 Udine, Italy; 23 Università di Padova, Museo di Zoologia, Via Jappelli, 1/a, I-35121 Padova, Italy; 24 Università di Pavia, Museo di Storia Naturale, Piazza Botta, 9, I-27100 Pavia, Italy; 25 Università di Catania, Dipartimento di Scienze Biologiche, Geologiche ed Ambientali, Via Androne, 81, I-95124 Catania, Italy; 26 Museo Civico di Scienze Naturali “E. Caffi”, Piazza Cittadella, 10, I-24129 Bergamo, Italy; 27 Natural History Museum, Cromwell Road, London, SW7 5BD, UK

**Keywords:** Biodiversity, Italy, metamuseum, natural history museums

## Abstract

The Italian natural history museums are facing a critical situation, due to the progressive loss of scientific relevance, decreasing economic investments, and scarcity of personnel. This is extremely alarming, especially for ensuring the long-term preservation of the precious collections they host. Moreover, a commitment in fieldwork to increase scientific collections and concurrent taxonomic research are rarely considered priorities, while most of the activities are addressed to public events with political payoffs, such as exhibits, didactic meetings, expositions, and talks. This is possibly due to the absence of a national museum that would have better steered research activities and overall concepts for collection management. We here propose that Italian natural history museums collaborate to instate a “metamuseum”, by establishing a reciprocal interaction network aimed at sharing budgetary and technical resources, which would assure better coordination of common long-term goals and scientific activities.

## Italy and biodiversity

Italy is universally known for its history, culture, food and art. Almost everyone knows the towns of Venice, Florence, and Rome, the classical Roman history which inspires architecture, literature and movies, and Leonardo da Vinci’s *Last Supper* and the *Mona Lisa*, which are among the most seen and reproduced paintings in the history of art. The list could go on for pages, but here we want to focus our attention on another invaluable and too often forgotten asset: natural history museums (NHMs) and the scientific specimens they preserve to document national (and international) biodiversity. A few numbers highlight the point: there are 12,000–13,000 species or subspecies of flowering plants in Europe, and approximately two thirds live in Italy. Furthermore, a rough count shows at least 160,000 animal species in Europe; in the recent Italian checklist their total for the country alone exceeds 56,000 (Minelli 1996).

## Museum legacy

Similar to what has happened elsewhere, many Italian naturalists of 19^th^ century, among which Orazio Antinori, Odoardo Beccari, Enrico Festa, Filippo De Filippi, Giacomo Doria, and Carlo Piaggia, visited remote areas of the world and documented biodiversity by collecting remarkable plant and animal specimens, which were deposited in Italian NHMs and became a great resource for studies and natural resources enhancement (Mazzotti 2011). NHMs act as the interface between science and the public, safeguarding scientific collections and promoting education (McCarter et al. 2001; Suarez and Tsutsui 2004). In fact, the scientific role of NHMs in life sciences is exceptionally relevant: to maintain and increase collections and to perform taxonomic studies. Taxonomy is indeed a science, which is particularly developed by curators, since type specimens – upon which the original descriptions are based – are usually deposited in their museum collections. Cataloguing world biodiversity is a specific museum mission, since this activity facilitates taxonomic activities and helps in protecting threatened species (Butler et al. 1998).

## A plea for natural history museums

We, the curators, taxonomists, science philosophers, and other members of the scientific community, are alarmed by the situation in which most Italian NHMs currently find themselves, with a continuing loss of scientific relevance, decreasing economic investments, scarcity of qualified personnel, and increasingly high risk for the long-term preservation of their collections. We wish to call urgent attention to this serious problem to relevant policy and decision makers. Unlike other countries (e.g., England, France, Spain, and USA), a national museum acting as the main repository for the larger part of these historical and contemporary natural history collections was never established in Italy. Because of this absence, in part due to historical reasons (Ruffo 2006), specimens collected during explorations and surveys were scattered throughout the country and deposited in different museums. The major museums (more than seventy according to the Italian Association of Scientific Museums, ANMS) vary in size and management type. Some are run by universities (e.g., Florence, Padua, Pavia, Perugia, Pisa, etc.), and others by public administrations (PAs) managed by local authorities, mainly municipalities (e.g., Genoa, Milan, and Rome), or by foundations (e.g., Venice).

## The importance of museums for taxonomic studies

While the existence of many scattered museums warranted until recently the material preservation of collections, it did not allow a proper development of the research component, which should accompany the constitution of scientific collections. In many cases university departments, which were the first to put together specimens and arrange natural history collections, do not consider museum-based research rewarding in terms of academic impact (also because papers dealing with traditional taxonomy rarely get a high citation index) and focus on functional disciplines, such as genetics, population biology, and ecology. This led to the wish to create new laboratories, often achieved by repurposing rooms housing old - and frequently rather dusty - zoological or botanical collections, which typically had been neglected for decades. In some cases the university museums were maintained, but they were more often used for practical classes with students or for public exhibits, and only in a few cases were they fully developed. At the same time, NHMs managed by local PAs were often more interested in public events with political payoffs for administrators, such as exhibitions, didactic meetings, and expositions than to collection-based researches.

## The relevance of research in museums

Scientific production is almost never considered as a parameter to evaluate the activity of curators in Italian NHMs. In general, the museum decision-makers appear to be not particularly focused on research activities carried out by their internal (curatorial) personnel. This is a striking difference with NHMs in other countries, where research represents a prominent and institutional product, which is evaluated regularly. As an example, most museums in Germany are autonomous research institutions often designated as “Forschungsinstitut”, as is the case with the Berlin, Bonn, and Frankfurt museums, and invest considerable economic resources into scientific activities, especially into management and implementation of reference collections and field-surveys in biodiversity hotspots. Nowadays, a commitment in fieldwork to increase scientific collections is not considered a priority by several Italian museums, and curators are rarely requested to carry out collecting campaigns or to study and catalogue biodiversity. In many cases they are only required to act as mere technicians in support of showy/public events, or as simple office-bearers following cultural and educational projects, while research is in most cases implicitly considered a secondary, time-consuming, and negligible activity. Although many curators tenaciously pursue their research line (mostly during their spare time), this is usually only possible in certain disciplines (such as entomology, malacology, and palaeontology), where taxonomy is still largely based on a morphological approach. On the other hand, laboratories with molecular tools and specialised technicians – nowadays quite commonplace in NHMs globally – are absent in Italian museums, thus seriously limiting capacities to carry out advanced biodiversity studies, to compete with foreign institutions, or to gain access to international funding.

## Foreign versus Italian museums

An analysis of *H*-values attained by NHMs’ curators showed that in other European countries (data from museums in Basel, Berlin, Bonn, Geneva, and the museums of the Senckenberg Gesellschaft) researchers affiliated to museums produce a higher number of indexed publications than in Italy (data from museums of Bergamo, Florence, Genoa, Milan, Padua, Pisa, Rome, Trento, Turin, Venice, and Verona), with a significant difference in *H*-index (9.96 ± 7.37 vs. 5.13 ± 5.11; Mann-Whitney text, p < 0.05). Despite hosting vast unique and invaluable collections, the absence of Italian NHMs from the group of institutions participating in synthesys, the European Union-funded integrated activities grant program, during the last ten years clearly betrays their management inadequacy (Bartolozzi 2013). The combination of recent economic cuts and loss of interest in scientific research by museum directions have brought the Italian NHMs to the brink of collapse and scientific irrelevance at the international level. Some museums have been closed to the public due to scarcity of funds and the difficulties in recovering from accidents: Udine has been closed since 1998 for renovation, that in Turin after an accidental explosion in 2013. Moreover, the number of curators working in these organisations has been dramatically decreasing for many years. While most European NHMs maintain a number of curators and technicians appropriate to their collection size (the curators at the Museum für Naturkunde in Berlin number approximately 30, while the figure for NHM in London exceeds 60), scientific personnel in Italian NHMs has become very insufficient or minimal, with some museums having the total scientific staff reduced to just one or two curators. During the last decade, retired curators have rarely been replaced and no recruitment has been undertaken for many years (in Genoa the last was in 1987, in Turin in 1991, and in Rome in 1993).

## The danger of losing collections

**Figure 1.**
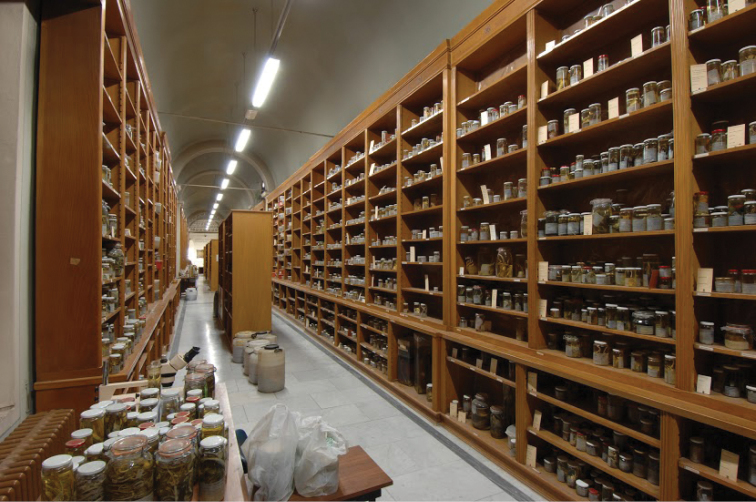
The herpetological gallery at the Museo di Storia Naturale, University of Florence (photograph by S. Bambi).

**Figure 2.**
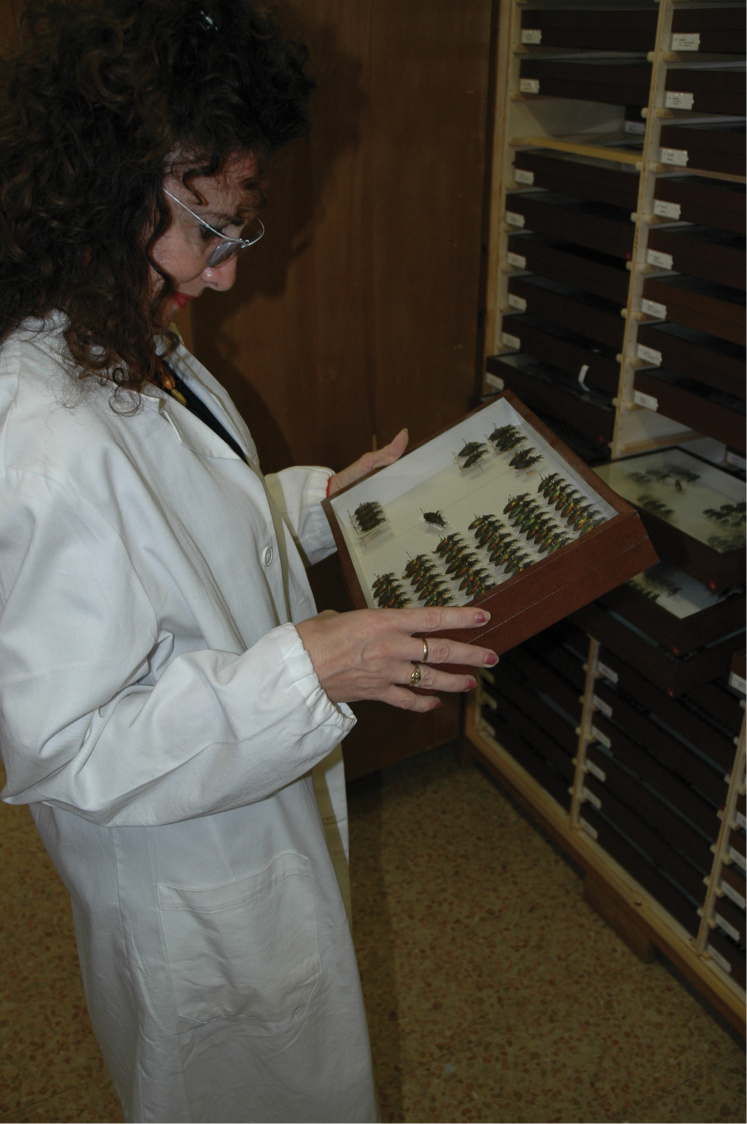
A technician caring at the entomological collection at the Museo Civico di Storia Naturale di Verona (photograph by L. Latella).

We consider such a situation extremely alarming, especially for ensuring the long-term preservation of natural history collections. The state of collections scattered among several museums (most with little interest in the scientific role of their materials) is inadequate and inappropriate. In particular, we are concerned about the impending demise of important collections: the number of type specimens housed in Italian museums is indeed considerable (at least 150 mammal taxa have their original types housed in Italian NHMs) and their conservation requires serious scientific preservation (Fig. 1). Sadly, most of these types are still uncatalogued, and this task cannot be done without assuring persistence and regular turnover and increase of the curatorial personnel. Moreover, as a result of this lack of personnel, basic technical tasks for daily management and educational activities have necessarily become priorities in many museums, forcing curators to redirect their activities, and to reduce or cease their research and assistance to other scientists (Fig. 2).

## Moving towards a metamuseum

We believe that the historical lack of a centralised museum has been detrimental for Italy. A large institution with a leading role and focused research could have facilitated scientific activity and political strategies on biodiversity as has happened elsewhere. The instigation of a centralised national museum is likely impracticable today, due to the fact that geopolitical conditions have changed. It is evident that scientific collections should be managed in a more efficient and unified way. Moreover, the Natural History Museum of Florence (one of the oldest in the world, dating to 1775), which owns some of the largest collections in Italy and maintains the old-time traditions of museum research in Italy, could be taken as an example and a possible repository of some national collections (Ruffo 2006, Gippoliti et al. 2014). An effective strategy is likely to be pursued through the association of most of the museums to form a sort metamuseum. This concept and idea were also reinforced on the occasion of a recent workshop held in Rome and organised by ANMS (the national association of natural history museums) and Accademia Nazionale delle Scienze, where the focus on scientific collections for research has been emphasised. The existing museums should establish a reciprocal interaction network, aimed at sharing budgetary and technical resources, assuring coordination of common long-term goals and scientific activities. The recent launch of CollMap (survey and mapping of the natural history collections) and “distributed institute of taxonomy” initiative by ANMS (Vomero 2013) is a further step in this direction. We suggest looking at the situation in Germany with the positive example of the Senckenberg Gesellschaft für Naturforschung and the Leibniz-Gemeinschaft, which demonstrate the connection between basic and applied sciences, integrating museums with universities, industries, and private partners. How this can be achieved is mostly a political matter, but cannot be postponed any longer and must urgently be integrated into the political agenda of the Italian government. For now, we hope that the MIUR (Italian Ministry for Education, University and Research) and the MIBACT (Italian Ministry for the Cultural Heritage and Activities and the Tourism) will soon pay long-overdue attention to our NHMs and adopt suitable policies for the safeguarding of their collections and the improvement of the associated research. At the same time, we are well aware that strong support must also be sought through European funds and pro-active collaboration of all interested partners.
